# Recent advances on bifunctional catalysts for one-pot conversion of furfural to γ-valerolactone

**DOI:** 10.3389/fchem.2022.959572

**Published:** 2022-08-09

**Authors:** Jianhua Wang, Zhiyan Xiang, Zexing Huang, Qiong Xu, Dulin Yin

**Affiliations:** National and Local Joint Engineering Laboratory for New Petrochemical Materials and Fine Utilization of Resources, Key Laboratory of Chemical Biology and Traditional Chinese Medicine, Ministry of Education, Hunan Normal University, Changsha, China

**Keywords:** furfural, γ-valerolactone, bifunctional catalysts, transfer hydrogenation, one-pot reaction

## Abstract

γ-Valerolactone (GVL) is one of the most valuable compounds derived from furfural (FAL), which has been industrially produced from agricultural byproducts like corn cobs. It is extremely challenging to synthesize GVL from FAL efficiently *via* a one-pot cascade reaction due to the need for multiple active sites in a single pot. By focusing on the aspects of one-pot synthesis of GVL from FAL, the authors aim to shed light on the rational design and utilization of environmentally friendly bifunctional catalysts with high efficiency in this reaction. Perspectives regarding future research opportunities in bi- or multi-functional catalysts for one-pot GVL synthesis are also discussed.

## Introduction

**FIGURE 1:**
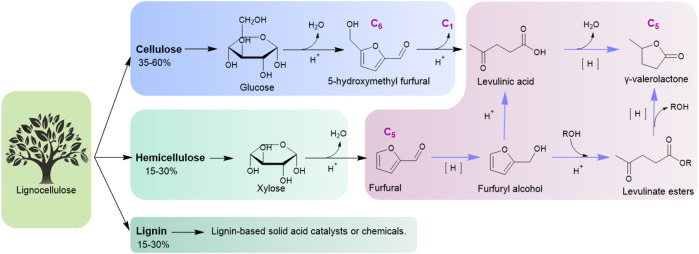
Production routes of GVL from lignocellulose.

In the 21st century, transition from a fossil-fuel industry to one based on renewable resources is a great challenge in the process of sustainable development (Jakob and Hilaire, 2015). Biomass is the only renewable carbon resource with a wide range of sources, abundant reserves, short formation cycles, and low prices, which can be used as an alternative resource for fuel and chemical production (Chua et al., 2019; Liu et al., 2020; Clauser et al., 2021). γ-Valerolactone (GVL) is an important intermediate compound derived from biomass. The unique physicochemical properties of GVL enables it to have a wide range of applications as fuel, fuel additive, polymeric material precursor, and green solvent (Alonso et al., 2013; Xu et al., 2020). Figure 1 shows the production routes of GVL from lignocelluloses (Raj et al., 2021). Lignocellulosic biomass, whose main components are cellulose/hemicellulose or derived platform compounds like 5-hydroxymethyl furfural (HMF)/furfural (FAL), can be converted into GVL smoothly.

Among these routes, the approach to producing GVL from FAL has attracted much attention. That is because first, it is a carbon-balanced process and has a good carbon-atom economy. In addition, FAL is non-edible, inexpensive, and can be produced from agricultural waste corn cobs industrially (Cui et al., 2016). This makes it possible to produce GVL on a large scale by a two-step reaction from lignocellulose to FAL and then to GVL. Therefore, great efforts have been made to one-pot conversion of FAL to GVL (Osatiashtiani et al., 2017; Yu et al., 2019).

There is a series of cascading reactions involving hydrogenation, ring-opening reaction, and lactonization in this reaction. Regarding the hydrogenation process for γ-valerolactone synthesis, metal catalysts such as Ru, Pt, and Ni (Nemanashi et al., 2018; Wang Y et al., 2020; Maumela et al., 2021) are efficient for direct hydrogenation using gaseous H_2_ as an H-donor in FAL hydrogenation and LA/levulinate ester lactonization. Compared with the use of H_2_ reduction, reaction systems using alcohol as an H-donor for one-pot production of GVL from FAL *via* a Meerwein–Ponndorf–Verley (MPV) reduction can be effectively catalyzed by acid catalysts (He et al., 2020; Antunes et al., 2022). As the ring-opening reaction of furfuryl alcohol (FOL) is also an acidic catalytic reaction, bifunctional catalysts with Brönsted/Lewis acid active sites have been greatly developed for the GVL synthesis *via* a MPV reaction (Osatiashtiani et al., 2017).

In this mini-review, the authors summarize the efficient bifunctional catalysts with metal/acid or Brönsted/Lewis acid active sites for one-pot conversion of FAL to GVL reported in the last 5 years. Table 1 lists the most efficient catalytic systems among them. Construction of acid centers as well as the supports’ structure are discussed, and catalytic reaction conditions are compared. It is hoped to provide insights into designing and developing efficient new sustainable catalysts for one-pot synthesis of GVL.

**TABLE 1:** Overview of important bifunctional catalysts for one-pot synthesis of GVL from FAL.

Entry	Catalyst	B/L acid ratio	Dosing ratio^a^	Optimum condition	GVL yield/%	Reference
1	Zr-HY + Al-HY	2^c^	2	120°C, 5 h, and 2-pentanol	85	Zhang T et al. (2019)
2	Chitosan-Ru/PPh_3_+HZSM-5	—	—	160°C, 30 h, and ethanol/formic acid = 95/5	79	Wang Y et al. (2020)
3	CuAl + HZSM-5	1^c^	0.5	160°C, 2 h, ethanol, and 5 MPa H_2_	90.5	Shao et al. (2021)
4	Zr-Al-beta (Al/Zr^b^ = 0.2)	0.05	1.2	170°C, 4 h, and 2-propanol	70.0	Melero et al. (2018)
5	Meso-Zr-Al-beta (Al/Zr^b^ = 0.77)	0.5	0.4	120°C, 24 h, 2-propanol, and 5% water	95.0	Song et al. (2017)
6	Hf-Al-USY	0.43	1.0	140°C, 12 h, and 2-propanol	64.9	Tang et al. (2021)
7	HPW/Zr-beta	0.31	2.5	160°C, 24 h, and 2-propanol	70.0	Winoto et al. (2019)
8	ZrO_2_-HPW-beta	0.65	1.3	170°C, 10 h, and 2-propanol	90.0	Rao et al. (2019)
9	ZrO_2_-Al-MFI-ns	0.035	2.5	170°C, 36 h, and 2-propanol	82.8	Kim et al. (2020)
10	Zr-CN/H-bate	—	2.2	160°C, 18 h, and 2-propanol	76.5	Zhang H et al. (2019)
11	HPW/ZrO_2_-SBA-15	0.49	1.31	170°C, 11 h, and 2-propanol	81.0	Rao et al. (2021)
12	Fe_3_O_4_/ZrO_2_@MCM-4	—	0.12	130°C, 30 h, and 2-propanol	85.7	Gao et al. (2021)
13	Sulfated DUT-67(Hf)MOF	—	0.4	180°C, 24 h, and 2-propanol	87.1	Li et al. (2019)
14	Meso-Al_2_O_3_-SO_3_H+0.1 g LiCl	4.35	1.33	120°C, 4 h, 2-butanol, ultrasonic power 90W, and duty cycle 60%	85.6	Karnjanakom et al. (2021)
15	FM-Zr-ARS	1.89	0.48	160°C, 8 h, and 2-propanol	72.4	Peng et al. (2021)
16	VPA-Hf	0.19	0.5	180°C, 14 h, and 2-propanol	81.0	Tan et al. (2022)
17	ZrP/HZSM-5	0.15	—	180°C, 10 h, 2-propanol, and 0.5 MPa N_2_	64.2	Ye et al. (2020)
18	Zr-P/SAPO-34	0.12	0.5	150°C, 18 h, and 2-propanol	80.0	Li et al. (2021)

a Furfural to catalyst mass ratio.

b Al/Zr molar ratio.

c Ratio of the two mixing catalysts.

## Efficient bifunctional catalysts

### Molecular sieve catalysts

Molecular sieves have regular pore structure, strong acidity, and high hydrothermal stability, which have been perceived as the most popular catalysts reported in the synthesis of GVL so far. Both physically mixed molecular sieve co-catalysts and modified bifunctional molecular sieve catalysts have been developed in one-pot synthesis of GVL from FAL. A physical mixture of Zr-HY and Al-HY zeolites was reported as a highly efficient catalyst system for the one-pot transformation of FAL to GVL using 2-pentanol as the hydrogen donor (Zhang H et al., 2019; Table 1, entry 1). GVL yields reached 85% only after 5 h of reaction at 120 C. The excellent activity of Zr-HY zeolites for the Meerwein–Ponndorf–Verley (MPV) reduction of FAL and levulinate ester was attributed to the larger pore size of HY-zeolites and stronger Lewis acidic sites. Also, Al-HY zeolites provided Brönsted acids, thus promoting furfuryl ether conversion to levulinate ester. Physically mixing H-ZSM-5 and metal catalysts was also reported as efficient metal/acid bifunctional catalytic systems in this reaction. Wang T et al. (2020) mixed chitosan-Ru/PPh_3_ and HZSM-5 in an ethanol/formic acid (95/5) solvent to catalyze the FAL one-pot cascade reaction (Table 1, entry 2). GVL yield was 79% after 30 h at 160 C. The unprecedented GVL yield of 90.5% has been reported in a dual catalyst system including CuAl and H-ZSM-5 in ethanol solvent and 5 MPa H_2_ (Shao et al., 2021; Table 1, entry 3). The relative abundance of the hydrogenation sites provided by CuAl and acidic sites provided by HZSM-5 determines the main products from furfuryl alcohol (FA) to ethyl levulinate (EL) or GVL. The biggest advantage of physically mixed catalysts is that the proportion and strength of acid sites can be independently optimized conveniently, thus avoiding complicated catalyst preparation. However, the physically mixed approach does not perform well when reusability and selectivity are concerned.

Since an appropriate proportion of Lewis and Brönsted acid plays a key role in the activity of the catalyst, molecular sieves with tunable Lewis and Brönsted acid sites prepared by doping metal ions or loading Brönsted acids have been widely reported (Li et al., 2020; Sun et al., 2022). Beta zeolite was treated in nitric acid solution and calcination was performed to remove Al, which can lead to more vacancies for the introduction of other metal species by post-impregnation (Melero, et al., 2018, Table 1, entry 4) or solid-state ion-exchange method (Song et al., 2017; Table 1, entry 5; Tang et al., 2021; Table 1, entry 6). The Zr(Hf)-modified molecular sieve catalysts showed superior catalytic activities in this reaction (Table 1, entries 4–10). In this type of dual acid site catalysts, Brönsted acidity is obtained from the Al sites and Lewis acidity is obtained from Zr or Hf sites. From the aspect of increasing catalysts’ Brönsted acid centers, phosphotungstic acid (HPW) has received attention for its strong Brönsted acidity (Frattini et al., 2017). HPW/Zr-beta and ZrO_2_-HPW-beta were prepared and applied in one-pot conversion of FAL in 2-propanol to give high GVL yields (Winoto et al., 2019; Table 1, entry 7; Rao et al.; Table 1, entry 8).

In addition to the effect brought by active sites, structure and morphology of the molecular sieve are also important for its catalytic efficiency. Taking ZrO_2_-[Al]MFI-ns 30 as an example, the MFI molecular sieve has a nano-sponge (NS) morphology, and its unique mesoporous structure allows the ZrO_2_ clusters (Lewis acid sites) to be confined within a few unit cell-thin porous silica-aluminate molecular sieve walls (Brönsted acid sites) (Kim et al., 2020; Table 1, entry 9). The generation of separate Brönsted and Lewis acid sites on the internal and external molecular sieves resulted in a significant enhancement of GVL yield (82.8%).

### Mesoporous silica catalysts

Mesoporous silica materials are good catalytic supports for their well-defined pore channels, large surface area, and tunable physical/chemical properties (Singh et al., 2018). ZrO_2_/SBA-15 and Zr-KIT were prepared by loading ZrO_2_ onto SBA-15 (Iglesias et al., 2018) and incorporating Zr into KIT-5 (He et al., 2019) and applied for GVL production by MPV reduction. A medium GVL yield of about 40% from FAL was reported. It may be because the acid strength and density of the catalyst is not enough.

Rao et al. (2021) reported a more efficient bifunctional mesoporous silica catalyst containing Zr and tungstophosphoric acid (TPA/ZrO_2_-SBA-15), which can completely convert furfural and give a GVL yield of 81%. (Table 1, entry 11). This research indicated that the acid–base properties of the catalyst were directed by the location of ZrO_2_ or TPA in the support, and that the catalyst with ZrO_2_ present inside the SBA-15 pores and TPA dispersed on the support showed the highest activity.

A magnetically recoverable multifunctional catalyst (Fe_3_O_4_/ZrO_2_@MCM-41) was tailored by the impregnation of ZrO_2_ supported on mesoporous MCM-41 coated with Fe_3_O_4_ nanoparticles for the cascade conversion of furfural (FAL) giving high yield of GVL. According to the literature, incorporation of Fe_3_O_4_ could not only impart the catalyst with a strong magnetism but also tune its acidity to promote GVL production (Gao et al., 2021; Table 1, entry 12).

### Other bifunctional catalysts

Among the most active bifunctional catalytic systems reported in the last 5 years, metal–organic frameworks (MOFs), porous Al_2_O_3_, and inorganic–organic framework catalysts are notable. As MOFs have a well-defined three-dimensional crystal structure, uniform active centers, high surface area, and porosity, they can be used as both catalysts and catalyst carriers. As shown in Table 1 entry 13, sulfated DUT-67(Hf) bifunctional metal–organic framework catalyst was prepared for the preparation of GVL reaching a yield of 87.1% (Li et al., 2019). The amount of Brönsted acid centers is regulated by adjusting the concentration of the aqueous sulfuric acid solution. But this catalyst cannot be recycled due to a dramatic loss of Brönsted acid sites in the reaction. A complex acid regeneration process was required before reuse.

A mesoporous Al_2_O_3_-SO_3_H catalyst was prepared by grafting sulfonic acid groups onto mesoporous Al_2_O_3_ (Karnjanakom et al., 2021, Table 1 entry 14). The mesoporous structure allows the Lewis and Brönsted acid sites in the catalyst to easily transfer intermediates and thus produces the desired GVL. The addition of salt (LiCl) increased the miscibility of the substrate and intermediates in the solvent phase and therefore increased the diffusion rate of the reacting molecules in the organic phase. It was also shown that ultrasonic assistance and the presence of O_2_ could inhibit the formation of humus and could maintain the stability of the catalyst. The catalyst could be reused for up to twenty runs without significant change. This catalytic system replaces the high-pressure hydrothermal system with a safer and greener ultrasonic/oxygen molecular system, which provides a new idea for the future one-pot method to produce GVL.

Peng et al. (2021) (Table 1 entry 15) reported a multifunctional Zr-containing catalyst (FM-Zr-ARS) with a stable porous inorganic–organic framework that showed high catalytic activity for GVL production (72.4% yield of GVL) from FAL (Table 1, entry 14). The -O-Zr-O- network in the FM-Zr-ARS structure formed a rich content of Lewis acid–base sites. The inherent sulfonic groups in ARS gave the FM-Zr-ARS hybrid an unsaturated acid site.

In addition, coordination organophosphate-Hf polymers (VPA-Hf) were prepared and found to exhibit superior performance in the one-step conversion of furfural to γ-valerolactone with a high yield of 81.0%, with a turnover frequency of 5.0 h^−1^ (Ye et al., 2020, Table 1 entry 16). The environmentally friendly material zirconium phosphate (ZrP) was also used as bifunctional catalysts in this reaction and showed good catalytic activity (Ye et al., 2020, Table 1 entry 17; Li et al., 2021, Table 1 entry 18).

It is noteworthy that ZrOCl_2_ as an acid/base bifunctional catalyst showed remarkable catalytic activity in the production of GVL (He et al., 2020). A maximum GVL yield of 63.3 and 52.1% was achieved from furfuryl alcohol and furfural at 200°C, respectively. [ZrO(OH)_2_]n• *x*H_2_O species and Brönsted acid species H^+^ were derived from *in situ* hydrolysis of ZrOCl_2_•8H_2_O. This research may provide a new idea for the construction of bifunctional catalysts.

## Conclusion and outlook

At present, researchers continue to explore various bifunctional or multifunctional high-efficiency catalysts for the one-pot production of GVL from FAL. To avoid the high pressure and high cost associated with direct hydrogenation (H_2_), alcohols, especially 2-propanol, are considered hydrogen donors for the production of GVL *via* MPV reduction reactions. In terms of bifunctional catalysts, molecular sieve catalysts with Brönsted/Lewis acid active sites are in mainstream GVL production. The highly active Lewis acid centers are mainly Zr or its cognate main group of Hf-based genera. Acting as Brönsted acids are Al-beta molecular sieves, H-ZSM-5 molecular sieves, HPW, zirconium phosphate, and sulfonic acid groups. The supports with a well-defined structure, well-developed pores, and an easily adjustable acid center will be more favorable for this reaction.

Though rapid development in developing efficient catalytic systems for one-pot conversion of FAL to GVL has been made, there are still some challenges. For example, humins are a big problem in the one-pot reaction, which decrease GVL yields and deactivate catalysts by deposition. Future research should also vigorously focus on exploring new bifunctional catalysts with high efficiency, low cost, energy saving, and environmental protection. Synergistic acid–base bifunctional catalysts can be developed to explore new catalytic systems. The plausible catalytic mechanism is worthy to be understood. Finally, one-pot synthesis of GVL from more upstream raw materials (e.g., lignocellulose, hemicellulose, or xylose) by using synergistic catalysis of Brönsted acid and Lewis acid is highly expected.
